# Intrathoracic stomach mimicking bone metastasis from thyroid cancer in whole-body iodine-131 scan diagnosed by SPECT/CT

**DOI:** 10.1590/2359-3997000000243

**Published:** 2017-01-27

**Authors:** Francisco Javier García-Gómez, Pablo Antonio de la Riva-Pérez, Cinta Calvo-Morón, Cristina Buján-Lloret, Teresa Cambil-Molina, Juan Castro-Montaño

**Affiliations:** 1 Department of Nuclear Medicine Virgen Macarena University Hospital Sevilla Spain Department of Nuclear Medicine, Virgen Macarena University Hospital, Sevilla, Spain

## Abstract

The whole-body iodine-131 scintigraphy is an imaging technique in monitoring patients with a history of thyroid cancer. Although the rate of false positives is negligible, it is not nonexistent. We report the case of an intervened and treated patient for thyroid cancer with good clinical and biochemical response. Scintigraphic findings were consistent with unsuspected bone metastasis. Fused SPECT/CT data allowed accurate diagnosis of giant diaphragmatic hernia associated with intrathoracic stomach, a very rare pathology that can lead to false positive results.

## CASE REPORT

**Figure 1:**
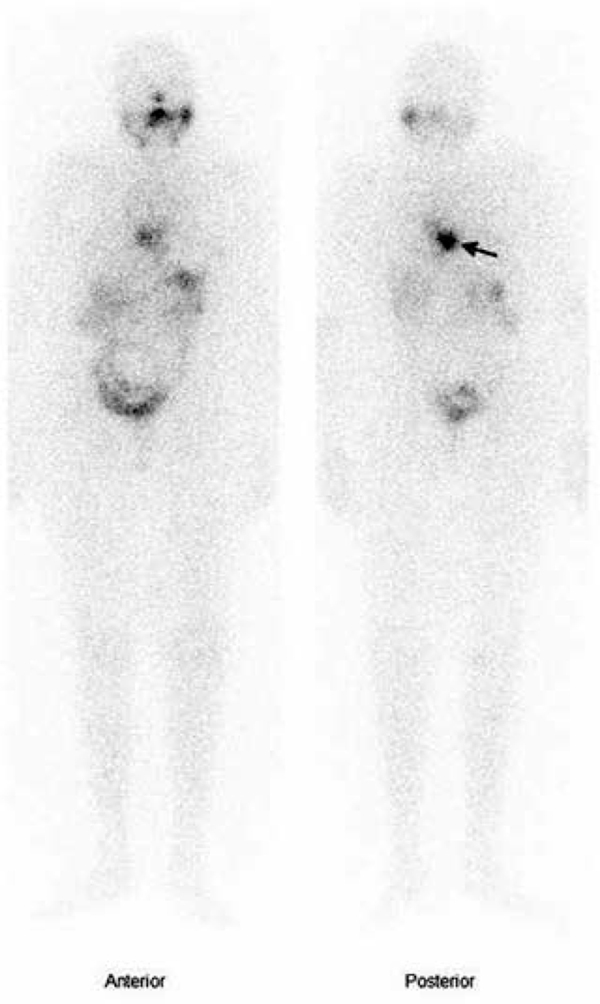
Whole-body iodine-131 scintigraphy. 150 x 252 mm (96 x 96 DPI).

We report a 76 year old female with history of T1N0M0 well differentiated papillary thyroid cancer being operated and treated with ablative dose of iodine-131 (^131^I). During regular follow-up, a whole-body ^131^I scintigraphy was performed 12 months after treatment ends in order to evaluate the persistence of thyroid remainders. Whole-body ^131^I scan ( Figure 1 ) revealed high intense hypermetabolic uptake foci, located in the midline. This finding was observed with greater intensity of uptake in the posterior projection ( Figure 1 , right panel; black arrow) being consistent with disease progression as spine bone metastasis. Intense back pain was referred when the patient was purposely questioned. Hormone levels at moment of scan were stimulated Tg < 0.2 ngr/mL, nonstimulated Tg < 0.2 ngr/mL and antithyroglobulin antibodies (TgAB) 349.4 U/L (Reference range: 0.0-200.0 U/L).

**Figure 2:**
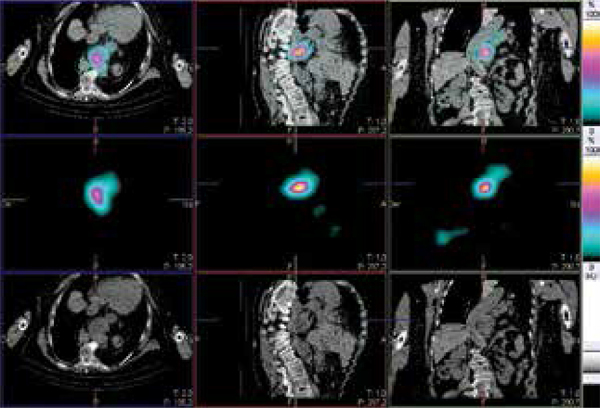
Axial, sagittal and coronal views (left, center and right columns) of hydrib iodine-131 SPECT/CT. Fused images are exposed in the upper row, the SPECT alone in the center row and CT alone in the lower row. 88 x 60 mm (300 x 300 DPI).

Subsequently, a single photon emission computed tomography/computed tomography (SPECT/CT) was performed due to discordance between biochemical findings (low hormone levels) and the clinical picture of intense back pain and functional imaging consistent with spine bone metastasis. A severe thoracolumbar scoliosis that determines a giant diaphragmatic hernia was revealed thanks to multimodality scan ( Figure 2 ). Thereby, high intense hypermetabolic foci corresponded to physiological activity of the improperly positioned gastric mucosa mimicking bone metastasis.

## DISCUSSION

The giant diaphragmatic hernia associated with intrathoracic stomach is a very rare entity ( 1 ) that seems to be related to increased intra-abdominal pressure and diaphragmatic laxity or kyphoscoliosis deviation in obese patients ( 2 ). Usually it corresponds to a type III hiatal hernia, with both sliding and paraesophageal components, wherein at least 30% of the stomach is in intrathoracic situation ( 3 ). Diaphragmatic hernias remain a diagnostic and surgical challenge, in which imaging techniques have become the cornerstone.

In another vein, whole-body ^131^I scintigraphy has proven to be a minimally invasive and safe technique that allows the diagnosis metastasis and recurrence after thyroidectomy when performed at 6-12 months of thyroid remainders ablation with radioiodine, as well as in monitoring during the long-term follow-up. However, the ATA and ETA guidelines state that low-risk individuals who have had a first post-radioactive iodine remnant ablation whole-body scan with an undetectable Tg level and a negative anti-Tg antibody level as well as a negative neck ultrasound do not require routine follow-up whole-body scan. Even if false positives in ^131^I body scans are extremely rare, they have been reported due to contamination by body fluids, ectopic thyroid tissue, infectious and inflammatory processes, benign and malignant tumors, serous cysts and even pulmonary bronchiolitis ( 4 , 5 ). Few cases of false negatives by intrathoracic hernia have been described, because the strange location of the physiological uptake of radioiodine by the gastric mucosa and the limited resolution of planar imaging may suggest that results from a recurrence of the disease ( 6 - 10 ). It can also be seen in healthy tissue, such as thymus, breast, liver and gastrointestinal tract ( 11 ) being able to be causes of false positive ( 12 ).

In this case, ^131^I SPECT/CT allowed reaching an accurate diagnosis, by discarding unsuspected metastatic bone disease and changing significantly the management and prognosis of the patient. Laparotomy or laparoscopic surgical repair is still the treatment of choice for giant hiatal hernia ( 13 ).

### Imaging findings

Whole-body scintigraphy after intravenous injection of 187 MBq (5 mCi) of ^131^I ( Figure 1 ) demonstrated physiological uptake of the radiotracer as well as a pathological uptake deposit which was located in the midline. This finding was present in both anterior and posterior projections, presenting much greater intensity in the latter (black arrow). Due to lack of precise anatomical definition of this technique, it was compatible with spinal bone metastases as a result of the central location, greater in backplanes and high intensity of uptake.

A ^131^I SPECT/CT (128x128 matrix; step and shoot mode; 360 degrees orbit; 25 seconds per image; low-dose CT: 140 kV, 2.5 mA) was performed in order to clarifying our clinical and radiological inconsistency. Fused images, SPECT alone and CT alone (top-down, respectively) revealed severe kyphoscoliosis associated with giant diaphragmatic hernia and intrathoracic stomach. In this way, hypermetabolic foci corresponded to physiological radiotracer uptake of the gastric mucosa.

It is known that SPECT/CT was highly accurate in patients who underwent a single challenge of radioiodine therapy ( 9 ). In our case, SPECT/CT helped clarify our diagnostic doubt and substantially modified the management and prognosis of the patient.
